# Lactylation Modification in Cardiometabolic Disorders: Function and Mechanism

**DOI:** 10.3390/metabo14040217

**Published:** 2024-04-12

**Authors:** Xu Li, Pingdong Cai, Xinyuan Tang, Yingzi Wu, Yue Zhang, Xianglu Rong

**Affiliations:** 1 Guangdong Metabolic Diseases Research Center of Integrated Chinese and Western Medicine, Guangzhou 510006, China; jsmjyyxg@outlook.com (X.L.); caipingdong2024@outlook.com (P.C.); tangxinyuan9917@outlook.com (X.T.); wyzgdpu@outlook.com (Y.W.); 2 Key Laboratory of Glucolipid Metabolic Disorder, Ministry of Education of China, Guangzhou 510006, China; 3 Guangdong Key Laboratory of Metabolic Disease Prevention and Treatment of Traditional Chinese Medicine, Guangzhou 510006, China; 4 Key Unit of Modulating Liver to Treat Hyperlipemia SATCM, State Administration of Traditional Chinese Medicine, Guangzhou 510006, China; 5 Institute of Chinese Medicine, Guangdong Pharmaceutical University, Guangzhou 510006, China

**Keywords:** lactylation, cardiometabolic disorders, heart failure, atherosclerosis, obesity, diabetes, hypertension

## Abstract

Cardiovascular disease (CVD) is recognized as the primary cause of mortality and morbidity on a global scale, and developing a clear treatment is an important tool for improving it. Cardiometabolic disorder (CMD) is a syndrome resulting from the combination of cardiovascular, endocrine, pro-thrombotic, and inflammatory health hazards. Due to their complex pathological mechanisms, there is a lack of effective diagnostic and treatment methods for cardiac metabolic disorders. Lactylation is a type of post-translational modification (PTM) that plays a regulatory role in various cellular physiological processes by inducing changes in the spatial conformation of proteins. Numerous studies have reported that lactylation modification plays a crucial role in post-translational modifications and is closely related to cardiac metabolic diseases. This article discusses the molecular biology of lactylation modifications and outlines the roles and mechanisms of lactylation modifications in cardiometabolic disorders, offering valuable insights for the diagnosis and treatment of such conditions.

## 1. Introduction

Cardiovascular disease (CVD) remains a prominent cause of death and impairment globally, despite advancements in healthcare, with numerous risk factors recognized [1]. Cardiometabolic diseases are a combination of risk factors associated with cardiovascular disease (CVD) and metabolic disorders such as diabetes mellitus, dyslipidemia, hypertension, and obesity. At the same time, the increasing cost of healthcare is placing a huge financial burden on patients and the healthcare industry. The search for new potent treatment approaches is critical, and it is becoming more and more crucial to explore the molecular pathways behind cardiometabolic diseases. The study of epigenetics has garnered a lot of interest in the biological sciences. Epigenetics, one of the key mechanisms of metabolic control in organisms, describes modifications in the expression of heritable genes based on variations in non-DNA sequences [2]. Post-translational modification (PTM) is a process wherein proteins undergo protein hydrolysis of shear groups (Serine, Cysteine, Aspartic acid, et al.) or covalent attachment to specific amino acid residues, resulting in the production of modifying groups. These modifications play a crucial role in influencing the function of proteins, ultimately affecting gene expression [3]. Since it was shown to have a crucial regulatory function in biological processes, lactylation has progressively grown in popularity as a topic of molecular biology study. Numerous research investigations have demonstrated that lactylation modification is a crucial aspect of post-translational modification and has been linked to various cardiometabolic disorders (CMDs) such as ischemic heart disease, heart failure, atherosclerosis, obesity, hypertension, and diabetes. The article provides a key target for future CMD therapy by summarizing lactate generation, metabolism, and the mechanism of lactylation and its role in the development of CMDs.

## 2. Cardiometabolic Diseases

Cardiovascular disease is the primary cause of illness and death globally, highlighting the urgent need for efficient and economical treatments to lower cardiovascular risk. Cardiometabolic disorders contribute to 31% of worldwide fatalities [4]. Cardiometabolic diseases are a cluster of multiple risk factors for cardiovascular disease and metabolic disorders. Metabolic illness and cardiovascular risk factors are closely interconnected. Dyslipidemia, hypertension, and insulin resistance are key components of the metabolic syndrome and significant risk factors for cardiovascular disease. These anomalies collectively contribute to the development of atherosclerosis and increase the risk of morbidity and mortality from cardiovascular disease. A single disease etiology, such as gene mutation or environmental exposure, cannot fully explain the origin of CMD, and the root causes of cardiometabolic illnesses and related disorders are not well understood. In recent times, significant progress has been achieved in the comprehension of cardiometabolic disorders. Next, we describe previous research advances in cardiometabolic diseases.

### 2.1. Ischemic Heart Disease and Heart Failure

Myocardial metabolism relies mostly on aerobic processes due to the limited glycogen reserves in cardiomyocytes, unlike other tissues and muscles. So, there needs to be a perfect balance between myocardial oxygen consumption (MVO2) and oxygen delivery in order to keep the ventricles working and protect the heart from ischemia [5]. Myocardial ischemia that results from atherosclerotic lesions’ obstruction or narrowing of the coronary arteries frequently results in ischemic heart disease. Ischemic heart disease primarily includes acute myocardial infarction and reperfusion injury. After myocardial infarction, early and effective restoration of blood flow to the heart muscle is crucial to reducing damage. This is achieved using either thrombolytic treatment or primary percutaneous coronary intervention (PPCI). Yet, the reperfusion process can trigger cardiac cell death and worsen myocardial tissue damage, known as myocardial reperfusion injury. There is presently no efficacious remedy for this ailment.

Heart failure is a progressive disease with an annual mortality rate of approximately 10% [6]. Heart failure is characterized by several alterations in the body, including modifications in the apoptosis of cardiomyocytes, the development of cardiac fibrosis, and alterations in gene expression levels. Various cardiac disorders, genetic abnormalities, and systemic diseases can lead to heart failure. Four main diseases, including ischemic heart disease, chronic obstructive pulmonary disease, hypertensive heart disease, and rheumatic heart disease, account for over two-thirds of all occurrences of HF. Heart failure prognosis remains poor despite rapidly evolving treatment [7].

### 2.2. Atherosclerosis

Atherosclerosis (AS), known for the yellowish look of lipids that build in the artery intima, is the most common among cardiometabolic disorders [8]. Atherosclerosis is a multifaceted pathological phenomenon that arises from a range of risk factors operating at distinct stages, encompassing endothelial dysfunction, lipid accumulation, proliferation and migration of vascular smooth muscle cells (VSMCs), the formation of foam cells, the development of plaques, the rupture of plaques, the occurrence of plaque hemorrhage, necrosis, calcification, and localized thrombosis [9]. Atherosclerosis is a chronic inflammatory condition affecting large and medium-sized arteries, leading to ischemic heart disease, strokes, and peripheral vascular disease. Thus, it is crucial to discover a remedy for atherosclerosis.

### 2.3. Obesity and Diabetes

Obesity is considered to be a distinct risk factor for the development of cardiovascular disease [10]. There is a clear correlation between obesity and the heightened incidence of morbidity and mortality in several chronic metabolic disorders [11]. In 1948, the World Health Organization officially classified obesity-related cardiomyopathy as a disease characterized by myocardial dysfunction or heart failure in obese individuals [12,13]. Obesity is commonly linked to an abnormal lipid metabolism. Extensive studies on obesity and energy balance have not been effective in addressing the obesity pandemic globally [14].

The correlation between obesity and type 2 diabetes mellitus is well-established and significantly contributes to the high incidence of type 2 diabetes mellitus in various nations. Type 2 diabetes is a significant risk factor for cardiovascular disease. Obesity is frequently linked to hypertension and dyslipidemia, resulting in a cluster of metabolic and cardiovascular risk factors in high-risk obese patients [15,16].

### 2.4. Pulmonary Hypertension

Hypertension, a condition characterized by elevated blood pressure, constitutes a significant risk factor for the development of cardiovascular and cerebrovascular diseases. The etiology of hypertension is multifactorial, including a complex interplay between genetic predisposition and environmental influences [17]. Pulmonary hypertension (PH) is a cardiovascular illness that has caused higher rates of disability and mortality in recent years. One of the notable pathological alterations observed in PH is the obstructive remodeling of the small pulmonary arteries. This remodeling primarily manifests as the gradual proliferation of pulmonary artery smooth muscle cells (PASMCs) [18]. As the disease progresses, it ultimately culminates in right ventricular failure [19,20]. There is insufficient effectiveness in preventing and treating PH. Research indicates that the development of hypertension may be affected by epigenetics, providing valuable information for therapeutic strategies [21].

## 3. Lactate Production, Transport and Shuttle

### 3.1. Lactate Production

Lactate has been mistakenly identified since its discovery as a metabolic waste product of hypoxic environments [22]. The glycolytic route is the primary source of lactate, a classic byproduct of glucose metabolism. Lactate is produced as fuel when the cell’s need for oxygen and ATP is greater than its supply, as occurs during intense exercise and illness [23,24]. Lactate can be classified as a hydroxyl carboxylic acid [25]. Because carbon atoms are not all symmetric, lactate is usually made up of three isomers: racemic DL-lactate, L-lactate, and D-lactate. D-lactate is an atypical metabolite present in tiny amounts within mammalian cells in a state of good health [26]. Consequently, many investigations have focused on L-lactic acid.

During aerobic glucose metabolism, pyruvate generated by glycolysis is converted to carbon dioxide and oxygen via oxidative phosphorylation, or OXPHOS [27]. In hypoxic circumstances, glycolysis catabolizes glucose to produce two pyruvate molecules. Then, lactate dehydrogenase changes pyruvate into lactic acid, which leads to the production of two adenosine triphosphate (ATP) molecules and one nicotinamide adenine dinucleotide (NADH) molecule at the same time [28]. The two primary subunits of LDH—LDHM and LDHH—are encoded by the LDHA and LDHB genes, respectively [29]. Mainly expressed in the heart, LDHB preferentially converts lactate to pyruvate. In contrast, mainly produced in skeletal muscle, LDHA preferentially transforms pyruvate into lactate [30]. In both anoxic and aerobic environments, lactate dehydrogenases (LDH) may catalyze the bidirectional conversion of pyruvate and lactate. Furthermore, glutamine catabolism in cancer cells can also result in the production of lactate [31].

**Figure 1:**
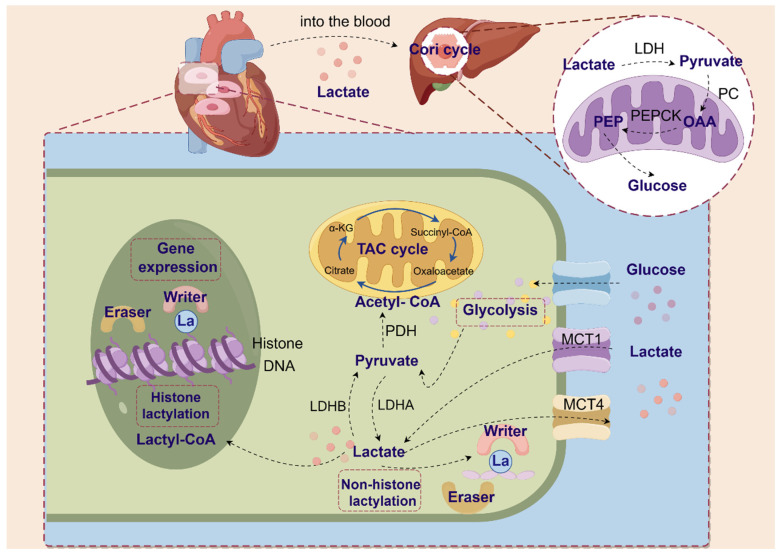
Shuttling of lactate through the cardiomyocyte and the mechanism of regulation of lactylation. Glucose is converted to pyruvate in cardiomyocytes via glycolysis. Pyruvate is then transformed into acetyl coenzyme A by pyruvate dehydrogenase to enter the TAC cycle. Pyruvate, with the assistance of lactate dehydrogenase, generates lactate. MCT1 simultaneously takes up lactate. Increased lactate levels stimulate the generation of lactyl coenzyme A and elevate histone lactylation, which in turn impacts gene expression. Elevated lactate causes lactate to rapidly enter the blood and enter the liver, where it is converted into glucose through gluconeogenesis.

While lactate serves a crucial function inside cellular processes, an excessive buildup of lactate can result in the condition known as lactic acidosis [32]. Lactate must thus be quickly digested and eliminated from tissues and circulation. The liver primarily eliminates lactate by producing ATP (oxidative phosphorylation, TCA cycle) and gluconeogenesis (Cori cycle). LDHB converts lactate to pyruvate, which pyruvate dehydrogenase (PDH) then uses to enter the TCA cycle as acetyl coenzyme A. This results in the irreversible elimination of lactate [22,33]. In addition, lactate is the major gluconeogenic precursor [34]. Lactate is catalyzed by LDH to pyruvate, which then enters the mitochondria and is converted to oxaloacetate (OAA) under the action of mitochondrial pyruvate carboxylase (PC). OAA is then catalyzed by phosphoenolpyruvate carboxykinase (PEPCK) to phosphoenolpyruvate (PEP), which is then shuttled to the cytoplasm for the gluconeogenic cycle to produce glucose, as shown in Figure 1 [35].

### 3.2. Transport and Shuttle of Lactate

The terms “intracellular” and “intercellular” in “lactate shuttle” refer to the roles that lactate plays in signal transduction and the supply of oxidative and gluconeogenic substrates [33,36]. The intracellular lactate shuttle encompasses the exchange of lactate between cytoplasm and peroxisomes [37] and mitochondria [38,39], whereas the cell-to-cell lactate shuttle involves the interchange of lactate between skeletal muscle and organs [40].

#### Monocarboxylate Transporters (MCTs)

There are three well-established mechanisms by which lactate is able to traverse a cell membrane: anion exchange, free diffusion, and transporters. Of these, transporters are primarily responsible for the lactate shuttle. Monocarboxylate transporters (MCTs) are an important class of proteins in cells with 14 isoforms (MCT 1-14 or SLC16A1-14). Together, they form a family called solute carrier family 16 (SLC16) [41]. MCTs are mostly made up of MCT1 and MCT4. MCTs are primarily responsible for transporting lactate across the cell membrane. MCT1 (SLC16A1) and MCT4 (SLC16A3) are the two main pathways for lactate entry into the cell and lactate efflux, respectively [27]. Lactate homeostasis in various tissues is largely dependent on the synergistic activity of MCT1-4 in physiological settings, which encourages the shuttle of lactate between glycolysis and oxidative cells [28]. Research has revealed that concentration gradients, pH gradients, and redox states all influence MCTs [23]. Because high-affinity MCT1 moves lactate based on the transmembrane lactate gradient, it is in charge of keeping normal tissues’ lactate levels stable. Low-affinity MCT4 transports lactate in cells with high amounts of lactate, such as tumor cells. The transport process begins with the binding of the free proton to MCTs. Subsequently, lactate binds to the MCT, leading to a conformational alteration inside the transporter protein. Consequently, the modified protein expels lactate on the opposite side of the membrane, concomitant with the liberation of the proton. Upon deprotonation, MCTs undergo a conformational alteration, ultimately reverting back to their original structure in anticipation of further transfer [28]. A lot of diseases, like symptomatic lactate transport deficit (SDLT), hyperinsulinemic hypoglycemia familial type 7 (HHF7), and monocarboxylate transporter 1 deficiency (MCT1D), are connected to MCT1 not working properly or not being expressed at all. Upregulation of MCT4 is common in cells that break down glycogen quickly and is strongly linked to the development of cancer [42].

### 3.3. Physiological Role of Lactate

Lactate assumes a significant function in diverse physiological processes inside the human body. Lactate is now recognized to have expanded beyond its previous designation as a metabolic waste product [43,44,45]. The primary fuel of the TCA cycle, lactate, greatly stimulates the TCA cycle and is necessary for the synthesis of energy [46]. The existing body of research indicates that lactate has the capacity to serve as an energy source [47,48], a signaling molecule [36,49], and an immunomodulatory molecule [50]. According to studies, circulating lactate helps to maintain energy balance throughout the body, serves as a stand-in for glucose as a source of energy for the brain, and satisfies excitatory brain activity when blood glucose levels are low [51,52]. Significant alterations in lactate levels have been observed in numerous disorders. First of all, lactate can regulate the tumor microenvironment, including promoting tumor-associated angiogenesis, promoting tumor growth and invasion, and generating immune escape. It is worth noting that another phenomenon known as metabolic symbiosis occurs when several cell types in the tumor microenvironment (TME) reuse lactate [53]. When cancer cells are not near the arteries and are exposed to low levels of oxygen, hypoxia-inducible factor-1α (HIF-1α) leads to an increase in the activation of genes that produce glucose transporters (GLUTs) and LDHA. It also promotes the uptake of glucose and the secretion of lactate and protons out of the cell through MCT4. Nevertheless, in cases where transformed cells are in close proximity to the blood vessels and have an adequate oxygen supply, lactate is absorbed by MCT1 and utilized as a source of energy following its conversion to pyruvate by LDHB [53].

In addition to providing energy to the tumor, lactic acid can also play a pro-inflammatory role. Localized lactate buildup typically coexists with acute inflammatory reactions or perfusion impairments. Lactate controls inflammation [54,55], improves ischemia-reperfusion injury [56,57], facilitates the body’s natural immune response within cells, and more [58]. Lactate amplifies inflammation in arthritic conditions by influencing immune cell activity and proinflammatory cytokine production in several ways [59]. M2-like macrophage polarization is facilitated by endothelial-derived lactate during ischemia, which aids in muscle regeneration [60].

Reactive oxygen species (ROS) generation is linked to the dysregulation of homeostasis in a variety of illnesses. Lactate can influence both enzymatic and non-enzymatic catalytic reactions that impact the cellular synthesis of ROS [61,62]. The primary fuel for mitochondrial respiration is lactate. Lactate is the primary electron donor and energy source for mitochondria, and its short-term cellular respiration increases the activity of the electron transport chain (ETC), which in turn increases the formation of ROS in the mitochondria [32,63]. Mitochondrial lactate metabolism generates hydrogen peroxide (H_2_O_2_), which further stimulates the generation of ROS [64].

#### Lactate Receptors GPR81 and GPR132

Lactate has the ability to transmit signals via G-protein-coupled receptors (GPCRs) [65]. In particular, lactate has the ability to generate signals via GPR81 (HCAR1) and GPR132 (G2A) [66], both of which are receptors sensitive to protons [67]. Inhibiting cAMP and protein kinase A (PKA)-mediated signaling, the lactate receptor GPR81, a G protein-coupled receptor that controls lactate transport between the plasma membrane and intracellular compartments, primarily inhibits lipolysis in adipocytes [68]. During intense activity, GPR81 is triggered to react quickly to elevated plasma lactate. However, GPR81 can also be activated by physiological amounts of lactate [68,69]. First, the lactate-GPR81 axis is involved in the regulation of inflammation during childbirth [70], inhibition of innate immunity [70,71,72], retinal function [73], neural activity [74], osteoblast differentiation [75], and intestinal [76], liver [71], renal [77], cardiovascular [78], and atherosclerotic [79]. Also, some types of tumor cell lines, like those from breast, colon, lung, hepatic, salivary gland, cervical, and pancreatic cancers, have high levels of GPR81 expression [80,81]. The regulation of lactate transport, angiogenesis, tumor formation, and metastasis in cancer cells is governed by GPR81 [74,80,81,82].

GPR132 is expressed in immune cells, particularly macrophages, in several organs, including the gastrointestinal tract and lung. Studies on lactate signaling via GPR132 have been few thus far. In mice that were fed a high-fat diet, L-lactate treatment decreased fat storage, insulin resistance, and infiltration of pro-inflammatory macrophages in adipose tissue without showing a connection to GPR132 [83]. Subsequent studies have found the GPR132-cAMP-PKA-AMPKα1 pathway, which is responsible for lactate signaling, causes macrophages to become anti-inflammatory when AMPK is activated [84]. Research in the Lewis lung cancer model showed that GPR132 is involved in promoting the pre-M2 phenotype in tumor-associated macrophages (TAMs) [85]. GPR132 is linked to M2 macrophages and metastasis in human breast cancer, but GPR132-KO mice do not experience metastasis [86]. To put it simply, the lactate receptor assumes a significant function in the metabolic processes of lactate, facilitating the connection between lactate signaling and the subsequent activation of pathways. This relationship is intimately associated with the progress of many pathological conditions.

## 4. Lactylation Is a Newly Discovered Post-Translational Modification

### 4.1. Discovery of Lactylation

Living systems are based on metabolic processes. In addition to having a common impact on metabolic activity, metabolic end-products and intermediates also serve as signaling molecules and carry out non-metabolic tasks inside metabolic activity. The acetylation process of histone lysine residues (Kac) is a good example. It starts with the intracellular metabolite acetyl-CoA, which is made during the tricarboxylic acid cycle [87,88]. Following the discovery of acetylation, two study groups discovered lysine lactylation (Kla), which consists of two types of Kla called L-lysine lactylation and D-lysine lactylation [89,90]. Zhang and his colleagues made the first proposal for L-Kla in 2019. L-Kla is a new type of PTM. The lysine residue of the core histone digested by trypsin from human MCF-7 cells showed a mass shift of 72.021 Da through high-performance liquid chromatography (HPLC)-tandem mass spectrometry (MS/MS) analysis. This was subsequently identified as L-lactate-derived histone lactylation (L-Kla) with the aid of the isotope L-lactate sodium (^13^C3) [89].

### 4.2. Non-Enzymatic Lysine Lactylation

It is important to note that the two versions of Kla vary in a few key ways, such as the target protein, the functional phenotype, and the source and chirality of the substrate. To begin, Zhang et al. thought that histone L-Kla would bind to L-lactyl-CoA, which is the activated form of L-lactate, when enzymes are active [89]. In contrast to Zhang et al.’s perspective, Gaffney and colleagues suggested a passive, non-enzymatic state in which the protein undergoes D-Kla alteration as a result of lactoglutathione (LGSH) being transferred to its lysine residue [90]. Glyoxalase 1 (GLO1) combines methylglyoxal (MGO), a by-product of glycolysis, with glutathione to make LGSH. GLO2 then hydrolyzes LGSH to produce glutathione and D-lactate [90,91]. Zhang et al. identified the presence of L-Kla on histones, highlighting that L-Kla is an epigenetically modified mechanism that plays a crucial role in controlling transcription [89]. Research has also found that L-Kla alterations are present on non-histone proteins. However, non-histone proteins—the majority of which are glycolysis-related enzymes—generate. Negative feedback control on glycolysis output is one of the primary targets of D-Kla alteration.

### 4.3. “Writer”, “Eraser” of Lactylation

Protein acylation is an evolutionarily conserved and reversible PTM [92]. Rapid and dynamic alterations in protein acylation have been shown to occur in response to enzymatic or non-enzymatic conditions [93,94,95]. In their study, Gaffney suggested that LGSH from MGO can directly lactylate lysine residues when acyl substitution is not done by an enzyme. Many lactylate lysines that were made when histone H4 was incubated with LGSH in vitro were confirmed [90]. However, many histone acylation processes involve effectors known as “writer” and “eraser”, which are responsible for adding and removing specific chromatin modifications, respectively. Certain types of acylation, such as acetylation, β-hydroxybutyrylation, succinylation, and lactylation, share two effectors: histone acetyltransferase and histone deacetylase (HDAC). These effectors are primarily involved in the acetylation or deacetylation functions [96,97,98].

The process of enzymatic lysine acetylation is reliant on the existence of lysine acetyltransferase (KAT)-mediated p300 and lactyl-CoA [89]. P300 is a classical histone acetyltransferase, which was first reported by Zhang et al. in 2019 to play the role of lactylation “writer”. In order to validate the role of p300 as a histone “writer” in cellular processes, the researchers conducted experiments involving the overexpression or knockdown of p300 in HEK300T cells. Subsequently, they detected a corresponding increase or decrease in the levels of histone Kla [99]. Following this, Yang et al. provided evidence for the involvement of P300 in lactylation. Previous research has shown that macrophages can take in extracellular lactate through MCTs and help lactylate HMGB1 through a process that depends on p300/CBP. If p300 or its homologue, CREB-binding protein (CBP), were turned off, lactylation of high mobility group box-1 (HMGB1) went down a lot [100]. Subsequently, Liu conducted a verification study in RAW264.7 cells, whereby p300 was found to function as a “writer, “influencing the activation of macrophages by modulating lactylation levels [101]. The investigations show that P300 functions as a “writer” and regulates the lactylation of lysine.

HDAC 1-3 have been recognized as novel “erasers” of H3K18la [102,103]. It is important to note that HDAC1-3 has the ability to remove both L-Kla and D-Kla. After more research using overexpression and knockdown experiments in cultured cells, it was found that HDAC1 and HDAC3, not HDAC2, play a specific role in delactylating histone Kla. Zessin et al. also said that HDAC2, HDAC3, HDAC8, SIRT2, and SIRT3 might be delactylate enzymes that work in vitro [104]. In a study, researchers discovered that SIRT3 functions as a site-specific eraser for H4K16la. They also made a chemical probe called p-H4K16la-NBD that lets them directly see the Kla delactylation process by showing it with fluorescence [105]. Although these studies have been reported as lactylation modifiers, these lactylation-modifying enzymes were primarily discovered and function in acetylation. Moreover, the current study has not found any “readers” for lactylation modification. The search for lactylation-specific modifying enzymes becomes an important direction.

### 4.4. Modification Sites of Lactylation

The phenomenon of lactylation was initially seen on histones, leading to subsequent investigations on the impact of lactylation specifically on histones. In a study conducted in 2019, Zhang et al. discovered 28 histone Kla sites present on core histones derived from human HeLa cells and mouse BMDM [89]. Following this, other research groups have used HPLC-MS/MS to report a multitude of additional histone Kla sites in various organisms, including mouse brain tissue [106], the protozoan parasite *Trypanosoma brucei* [107], *Botrytis cinerea* [108], rice [109], and maize [110]. In their study, Hagihara et al. conducted an investigation of the prefrontal cortex of mice with reduced social behavior. The researchers successfully discovered 16 histone Kla loci in this brain region. Notably, they observed an upregulation of histone H1 lactylation, which appeared to be a physiological reaction to the stress induced by social failure [106]. In their study, Zhang et al. found that *Trypanosoma brucei* has 16 histone Kla sites spread out in both normal and variant histones. Notably, the authors observed that all 16 Kla sites exhibited associations with other PTMs [107]. Additional research groups of interest are those investigating *Botrytis cinerea* and rice, which have successfully found six and fourteen histone Kla sites, respectively [108,109]. Shi and colleagues first found 37 Kla sites on 16 histones in the maize genome and observed clear differences in histone Kla patterns between drought-sensitive (B73) and drought-tolerant (Jing2416) lines using ChIP-Seq [110].

In addition to the core histones, high-throughput techniques like HPLC-MS/MS have made it easier to find many Kla sites on different proteins found in different parts of cells, such as the nucleus, cytoplasm, mitochondria, ribosomes, and cell membranes, as shown in Table 1. In 2020, Gaffney et al. discovered 350 proteins in HEK293 cells that had been changed by lactoyl lysine. Most of these proteins were enzymes that help break down glucose [90]. In the study conducted on B. cinerea, a comprehensive analysis revealed the presence of 166 Kla sites within a pool of 273 lactated proteins [108]. Additionally, Zhang et al. conducted an analysis on T.brucei and discovered 387 Kla sites from a pool of 257 proteins [107]. Meng and others also reported similar findings, showing 638 Kla sites in 342 proteins in rice grains [109]. In 2022, gastric cancer (GC) cells were analyzed and it was found 2375 Kla sites, showing a notable enrichment of these proteins in spliceosome function [111]. Zhao et al. discovered 983 lysine lactylation sites on 523 proteins in whole protein extracts from *Toxoplasma gondii* in 2022 [112]. In 2022, An et al. identified 1458 Kla sites in 469 proteins from *Frankliniella occidentalis* through a comprehensive investigation of the lactylome in the species [113]. Yin et al. discovered 1964 Kla sites on 955 proteins in the T. gondii RH strain [114]. Song et al. conducted a comprehensive investigation of the global lactylome in *Phialophora verrucosa* and identified a total of 636 lysine lactylation sites on 420 proteins [115].

In 2023, Lin performed the initial comprehensive Kla analysis on tendon samples taken from patients with rotator cuff tendinopathy (RCT), revealing 872 Kla sites in 284 proteins [116]. Wang also identified a total of 868 lactlyation sites on 379 proteins in the marine N. oceanica strain [117]. Wu et al. identified 215 lysine lactylation sites in 139 modified proteins in sugarcane [118]. Wu conducted a lactylome analysis to study the role of Kla in hepatocellular cancer (HCC) by collecting three normal liver samples, three HCC samples (without metastasis throughout a 3-year follow-up period), and three lung metastatic samples of HCC. A total of 2045 Kla sites were discovered on 960 proteins [119]. Wang and colleagues studied the lactylome of retinal microglia in low-oxygen conditions. They found 3093 Kla sites on 751 proteins, with 77 sites on 67 proteins showing elevated Kla levels under hypoxia [120]. A study conducted in 2023 examined Kla sites in human lungs under normal physiological settings and discovered 724 Kla sites on 451 proteins [121]. An amount of 1003 Kla sites were discovered on 469 proteins in a study evaluating the Kla of cortical proteins in a Rattus norvegicus model of cerebral ischemia–reperfusion injury (CIRI) [122]. After that, Yang et al.’s combined lactylome and proteome analysis of HCC tumors and nearby livers found 9275 Kla sites, including 9256 sites on non-histone proteins, which suggests that Kla is a widespread modification [123]. Cheng and coworkers discovered 637 lysine lactylation sites in 444 proteins in FHC and SW480 cells [124].

In addition to the aforementioned high-throughput mass spectrometry (MS) investigations, other scholarly inquiries have reported the presence of lactylated proteins. In a separate investigation conducted by Xiong et al., two specific Kla sites inside methyltransferase 3 (Mettl3) were found. These sites, namely K281 and K345, are situated in distinct regions of the protein. Specifically, K281 is located within the first CCCH-type zinc finger domain, while K345 is positioned within the adaptor region [125]. Gu et al. showed that lactate can promote the lactylation of MOESIN at K72 to control the formation and immunosuppressive role of Regulatory T (Treg) cells [126]. Enzymes involved with metabolic processes, particularly glycolysis, are frequently changed by Kla. Fructose bisphosphate aldolase A (ALDOA) is lactylated at residue K147, leading to a reduction in ALDOA enzymatic activity. This may provide a negative feedback mechanism, allowing cells to lower high lactate levels [127]. At the same time, α-enolase, a glycolytic enzyme, is lactylated at K343, perhaps influencing interactions between substrate and enzyme. Dehydrogenase reductase 7, an enzyme with broad substrate specificity for steroid hormones, lipids, and other metabolites, can undergo lactylation at K321 [127]. The centromere proteins (CENPs) are essential protein complexes that play a crucial role in kinetochore formation and chromosomal segregation during mitosis. Liao and colleagues discovered that CENPA was markedly increased in HCC, and its high expression was associated with a worse prognosis in HCC patients. CENPA was shown to be lactylated at lysine 124 (K124) [128]. Chen showed that MRE11, an important protein involved in homologous recombination (HR), is modified by lactylation at K673 by the CBP acetyltransferase in reaction to DNA damage, and this process relies on ATM phosphorylation [129]. Meng employed LC-MS/MS to confirm certain modification sites in the DCBLD1 protein, revealing that DCBLD1 is a substrate for lactylation, with the major lactylation site located at K172 [130].

Additionally, it was found that some proteins undergo lactylation, but their lactylation sites were not revealed. Ricardo A. Irizarry-Caro et al. found that the TLR signaling adapter BCAP regulates histone lactylation and thus macrophage transformation, but the histone modification sites were not elucidated [131]. Yang and colleagues discovered that the nuclear protein Hmgb1 can undergo direct lactylation via a p300/CBP-dependent process when exposed to lactate, causing it to move from the nucleus to the cytoplasm [100].

Furthermore, the precise localization of Kla plays a crucial role in comprehending the chemical mechanism underlying lactylation. To address this, Jiang et al. introduced a web-based tool called FSL-Kla, which serves as a predictive model for determining the position of Kla [132]. In 2023, Lai and Gao introduced a precise and efficient prediction approach named Auto-Kla, which utilizes automated machine learning to effectively differentiate Kla sites [133].

## 5. Lactate Metabolism, Lactylation, and Cardiometabolic Disorders

The heart has a significantly high energy demand and necessitates the continuous production of substantial quantities of ATP to sustain its contractile capabilities [134,135]. In the absence of conversion, the heart will experience ATP depletion within a timeframe of 2 to 10 s, resulting in systolic failure. Lactate serves as a significant energy source for the heart, particularly when there are increased amounts of lactate in circulation. It facilitates the fulfillment of cardiac ATP demands by engaging in mitochondrial oxidative phosphorylation metabolism [136,137]. Furthermore, it has been proposed that lactate may possess signaling capabilities [138] and, under certain circumstances, contribute to the TCA cycle in a pyruvate-dependent fashion [139,140]. Research has demonstrated that both lactate and lactylation modifications have a significant impact on cardiometabolic disorders, as shown in Table 2.

### 5.1. Ischemic Heart Disease and Lactylation

**Figure 2:**
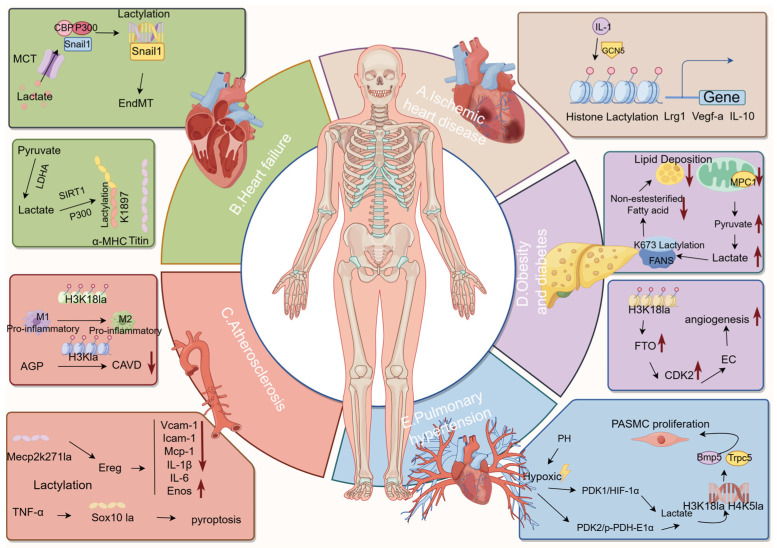
Primary mechanism of action of lactylation modifications in cardiometabolic diseases. The image highlights five aspects of cardiometabolic disease: ischemic heart disease, obesity and diabetes, hypertension, atherosclerosis, and heart failure. It illustrates the regulatory processes of histone and non-histone lactylation for each area.

Recent research has indicated that the biological effects of lactate can be attributed to protein lactylation, a process in which lactate modifies certain lysine residues of histone or non-histone proteins, hence altering their activities after translation [89,123,141]. The research by Wang et al. demonstrated the significance of histone lactylation in controlling the dual anti-inflammatory and pro-angiogenic functions of monocyte macrophages. The researchers established that histone lactylation promotes the transcription of reparative genes, hence creating a favorable environment for tissue repair. Furthermore, this study demonstrated that IL-1β-dependent GCN5 recruitment catalyzes the lactylation of protein H3K18, thereby improving cardiac function after myocardial infarction [142]. Their research found the monocyte-histone lactylation regulatory mechanism that happens after a myocardial infarction and how it controls the expression of repair genes that follow, as shown in Figure 2A.

### 5.2. Heart Failure and Lactylation

Lactate was formerly perceived as a metabolic by-product; nonetheless, it holds significance as a crucial energy source for cardiac function. Lactate equivalents and sodium lactate therapy have been observed to provide relief for acute heart failure in both animal trials and clinical research [143,144]. Empirical investigations have demonstrated that elevated lactate concentrations are prevalent among individuals diagnosed with heart failure, and such elevations have been linked to increased mortality rates in this patient population [145]. In their study, Fan et al. discovered that Snail1 lactylation had a significant role in facilitating the advancement of cardiac fibrosis subsequent to myocardial infarction [146]. Subsequent research has provided more evidence indicating that the presence of lactate promotes the interaction between CBP/p300 and Snail1, resulting in the formation of Snail1 lactylation, which is a transcription factor associated with TGF-β.

Fan confirmed the relationship and mechanism of lactylation in post-infarction cardiac fibrosis, and Zhang et al. subsequently demonstrated the significant significance of particular lactylation alterations in heart failure. Lactylation of the alpha-myosin heavy chain (alpha-MHC) lysine 1897 site encoded by the Myh6 gene is reduced in mice and heart failure patients. Mice with a knockout of the α-MHC K1897 gene demonstrate compromised interaction between α-MHC and Titin, leading to the manifestation of severe heart failure [147]. Excessive lactate efflux and depletion in cardiomyocytes after heart damage led to lower intracellular lactate levels, reduced α-MHC K1897 lactylation, and α-MHC-myosin interactions. This confirmed the significance of lactylation in heart disease, as shown in Figure 2B.

### 5.3. Atherosclerosis and Lactylation

Vascular smooth muscle cells exhibit a remarkable degree of plasticity, enabling them to transition from a contractile phenotype to a proliferative phenotype in the context of atherosclerosis. Yang et al. found that the presence of lactate can trigger the transition of VSMCs from a contractile phenotype to a proliferative phenotype, which plays a role in the pathogenesis and treatment of atherosclerosis [148]. This phenotypic switch facilitates the migration of VSMCs towards the intima, hence contributing to the development of intimal hyperplasia and subsequent stenosis. Pyroptosis is a type of cellular death that exhibits the morphological and pathophysiological features of both apoptosis and necrosis. The phenomenon is distinguished by the fast development of holes in the plasma membrane, necrosis resulting from osmotic cell swelling, and the discharge of a substantial quantity of cellular constituents and pro-inflammatory agents. Research has demonstrated that LDHA can trigger pyroptosis in cerebral ischemia/reperfusion damage (CI/R) through histone lactylation xx [149]. Xu et al. further verified the modulation of pyroptosis by lactylation. Sex-determining region Y (SRY)-related HMG-box gene 10 (Sox10) was demonstrated to have a vital role in promoting vascular inflammation by engaging in the transformation of vascular smooth muscle cells and triggering pyroptosis in neointimal hyperplasia. The main process involves a transcriptional program of transdifferentiation induced by tumor necrosis factor-α (TNF-α) through the promotion of sox10 lactylation [150]. Neointimal hyperplasia plays a crucial role in atherosclerosis development, suggesting that pyroptosis may be the mechanism via which lactylation influences atherosclerosis.

Throughout the course of atherosclerosis, the extravasation of circulating immune cells into organs is regulated by endothelial cells (ECs) through the secretion of cytokines, chemokines, and adhesion factors. Lactate has been found to induce the activation of signaling pathways inside endothelial cells, hence exerting a regulatory influence on the inflammatory response [151]. First, Wang and colleagues discovered that the process of histone lactylation plays a role in regulating the dual activities of monocyte macrophages, specifically their anti-inflammatory and pro-angiogenic functions [142]. Research has demonstrated that exercise is a reliable and effective method for both preventing and mitigating the progression of cardiometabolic illnesses. In their study, Wang et al. discovered that exercise training had a positive impact on the promotion of Mecp2 lysine lactylation (Mecp2k271la), hence inhibiting the progression of AS. Specifically, Mecp2k271la repressed the expression of extra-epitope regulatory protein (Ereg) by binding to its chromatin, demonstrating that Ereg functions as a significant downstream molecule of Mecp2k271la. Then, Ereg modified the signaling pathway of mitogen-activated protein kinase (MAPK) by regulating the level of phosphorylation of the epidermal growth factor receptor. This regulation subsequently influenced the expression of Vcam-1, Icam-1, Mcp-1, IL-1β, IL-6, and Enos in ECs, ultimately contributing to the regression of atherosclerosis [152].

Macrophages that have undergone activation can be categorized into two distinct phenotypes based on their polarization status. M1 macrophages exhibit pro-inflammatory characteristics, characterized by their tendency to engage in aerobic glycolysis and produce ATP and lactate. Additionally, M1 macrophages release a significant quantity of pro-inflammatory factors, including TNF-α, IL-6, and nitric oxide synthase (iNOS). On the flip side, M2 macrophages secrete a substantial number of anti-inflammatory factors such as IL-10, arginase-1(Arg1), and IL-13, which primarily contribute to tissue remodeling processes. Research has demonstrated that lactate has the ability to inhibit the inflammatory responses mediated by macrophages in an environment characterized by both hypoxia and inflammation [148,153]. Macrophages are the predominant immune cell population within atherosclerotic plaques and are widely believed to exert a pivotal influence on the immune response and advancement of atherosclerosis [154]. The study conducted by Zhang et al. showed that the accumulation of lactate in metabolic processes stimulates the activation of M1-type macrophages, subsequently leading to the initiation of endogenous lactate synthesis. Endogenous lactate promotes histone lactylation modification and linkage to the macrophage genome, metabolic reprogramming, induces the conversion of M1-type macrophages to M2-type macrophages, and promotes the expression of genes related to tissue repair, such as Arg1 [89]. The process of lactate modification of histones may provide an explanation for the underlying mechanism via which macrophages transition from an M1 to an M2 phenotype. Research by Irizarry-Caro and colleagues has demonstrated that BCAP, a signaling adaptor linked to Toll-like receptors (TLRs), decreases glycogen synthase kinase 3 (GSK3) and forkhead box protein O1 (FOXO1) activity and regulates the transition of macrophages from an inflammatory to a reparative phenotype by facilitating histone lactylation [131]. Lipopolysaccharide (LPS) has the ability to stimulate the expression of long non-coding RNA (lncRNA) and cause an increase in the levels of LINC00152 in macrophages, resulting in the suppression of pro-inflammatory cytokine expression. LPS upregulates the expression of LINC00152 and reduces the binding efficiency of the inhibitory factor YY1 to it through the introduction of histone lactylation at its promoter, thus revealing a potential mechanism by which histone lysine lactylation regulates the inflammatory response [155].

The secretion of accumulated lactate in atherosclerosis occurs through the utilization of pumps, exchangers, and lactate transporter proteins, which effectively decrease the extracellular pH [156]. Whereas low extracellular pH affects the process of lipid accumulation [157,158,159]. In addition, studies have provided evidence indicating that lactate results in a reduction in pH levels, therefore causing a decline in both the quantity and functionality of HDAC within the nucleus [160]. Atherosclerosis encompasses aortic valve calcification. Moreover, aortic calcification is strongly associated with aging, hypertension, and disorders linked to glycolipid metabolism. Andrographolide (AGP) effectively reduced calcium deposition in valve interstitial cells (VICs) and improved aortic valve calcification. Wang and colleagues showed that AGP reduces calcification by disrupting H3Kla through p300 [161]. Research has demonstrated that lactylation modification plays a role in the development of atherosclerosis, making it a significant focus in atherosclerosis research, as shown in Figure 2C.

### 5.4. Obesity, Diabetes, and Lactylation

In contrast to glucose, the oxidation of fatty acids produces approximately 10 times more ATP per molecule. Hence, organs characterized by high energy demands, such as the heart and kidneys, predominantly rely on circulating lipids as a source of energy. Lipoproteins, comprising triglycerides, cholesterol, phospholipids, and several other lipids, have a significant function in the process of circulation. Nevertheless, hypercholesterolemia stands out as the prevailing form of dyslipidemia, with high levels of low-density lipoprotein cholesterol (LDL-C) serving as a significant contributor to the risk of cardiovascular disease. Hence, the investigation of aberrant lipid metabolism has emerged as a significant area of research in the field of cardiovascular metabolic diseases.

The rise in worldwide obesity rates has correspondingly led to an increase in the prevalence of metabolic disorders. The liver serves as the primary organ responsible for lipid metabolism, and its metabolic disorders are intricately linked to the condition of obesity. The mitochondrial pyruvate carrier (MPC) is a crucial carrier protein situated on the inner membrane of the mitochondria [162]. It consists of two subunits, both of which play a physiological role in transporting pyruvate produced by glycolysis to mitochondria for downstream metabolic processes such as the TCA cycle [163]. MPC has been shown to be a regulator of organ metabolism, including the liver, adipose tissue, and heart. The study conducted by Gao et al. demonstrated that the knockdown of MPC1 in hepatocytes had an impact on fatty acid synthase through the regulation of lactate levels. The inhibition of fatty acid synthase activity and the reduction of hepatic lipid deposition were seen upon lactylation of the K673 site of fatty acid synthase [164].

In a subsequent study, Chen et al. observed a notable impact of high-intensity interval training on the modulation of lipid metabolism. High-intensity interval training (HIT) exhibited a notable increase in the up-regulation of protein lactylation in inguinal white adipose tissue (iWAT), with fatty acid synthase (FASN) displaying the highest number of modified sites. The application of lactate in 3T3-L1 cells resulted in an elevation in FASN lactylation, a suppression of FASN activity, and a reduction in the synthesis of palmitate and triglycerides [165]. Hence, Chen’s findings present compelling data supporting the role of exercise in enhancing lipid metabolism and consequently mitigating metabolic heart disease. In addition, Huazhuo Tiaozhi Granule (HTG) is a widely used herbal formula with lipid-lowering effects in clinical practice. HTG has been shown to have a notable impact on blood lipid profiles as well as the accumulation of lipids in the liver. Yin et al. demonstrated that lactylation was observed in 198 proteins after HTG treatment, including histone H2B (K6) and H4 (K80) lactylation. HTG promoted lactylation in hepatocytes, further validating lactylation modification as one of the important targets for regulating lipid metabolism [166].

Elevated fasting lactate levels have been observed in individuals with obesity and type 2 diabetes [167,168,169], and these levels have been found to be correlated with insulin resistance [170]. The skeletal muscle is a significant contributor to lactate and plays a vital role in maintaining glucose homeostasis. It is estimated that around 70–80% of glucose uptake, which is regulated by insulin, takes place in the skeletal muscle [171]. In their research, Maschari et al. utilized clinical samples obtained from both lean and obese individuals to provide empirical evidence supporting the existence of lactylation alteration in human skeletal muscle. The concentration of lactate in the body is correlated with the presence of circulating lactate and the development of insulin resistance in individuals who are fat [172]. Diabetic retinopathy (DR) is one of the complications of diabetes that causes irreversible vision loss. Diabetes leads to the overexpression of FTO by lactate-mediated histone lactylation, which impacts endothelial cells by controlling CDK2 mRNA stability in a m6A-YTHDF2-dependent way, promoting angiogenesis in several experimental models. FTO controlled communication between endothelial cells and pericytes to cause leakage in diabetic microvessels and facilitated contacts between endothelial cells and microglia to promote inflammation and neurodegeneration in both living organisms and laboratory settings [173]. Metformin, a classical medicine for diabetic therapy, decreases histone (H3K18) lactylation, resulting in reduced ROS generation and a weakened neutrophil response to injury and inflammation [174]. The investigation into lactate and lactylate modification in relation to obesity and diabetes indicates that lactylation modification holds significant promise in the realm of obesity disorders, particularly those associated with obesity and diabetes, as shown in Figure 2D.

### 5.5. Pulmonary Hypertension and Lactylation

Abnormal glycolytic switching at the molecular level of PH has attracted much attention [175,176,177,178]. The role of mitochondrial reactive oxygen species (mROS) as a significant stress mediator has been recently recognized in its capacity as a sensor of hypoxia during episodes of PH [179,180,181]. The study conducted by Chen et al. demonstrated that in conditions of reduced oxygen availability (hypoxia), there was an observed rise in mROS levels. This increase in mROS was shown to be associated with coordinated metabolic shifts towards glycolysis and the proliferation of PASMCs. Furthermore, the researchers determined that this process was dependent on the presence of hypoxia-inducible factor 1-alpha (HIF-1α). Lactate, which is a target of HIF-1α and is mechanistically linked to cell proliferation, facilitates the process of histone lactylation, specifically involving H3K18la and H4K5la [182]. Research has discovered that when lactate production is decreased, it hinders the modification of lactylation and thus restricts the formation of PASMC and vascular remodeling [182]. The process of histone lactylation, which is a recently discovered epigenetic change, presents a promising route for the development of therapeutic interventions in the context of PH.

Research has shown that histone lactylation influences gene expression, including m6A methylation-related enzymes. The expression of enzymes is implicated in the regulation of pulmonary arterial hypertension. The presence of lactate within the intracellular milieu facilitates the process of lactylation on histone H3 pairs located on the promoters of homeostatic genes, thus leading to the activation of their expression. Multiple studies have provided empirical evidence supporting the enrichment of H3K18la within the promoter region of METTL3. The transcription of METTL3 is facilitated by lactate through the modification of H3K18la [125]. The expression of METTL3 is increased in PASMC under hypoxic conditions, leading to the promotion of remodeling in the pulmonary artery [183]. The regulation of histone lactylation is influenced by the metabolic dynamics of glucose and lactate levels. Prior research has demonstrated the enrichment of H3K18la at the promoter region of YTHDF2 and the consequential regulation of YTHDF2 transcription by H3K18la [184]. The protein YTHDF2 has been seen to decrease the process of translation and the level of expression of LDHB. Additionally, YTHDF2 has been found to impede aerobic glycolysis and the proliferation of cells by facilitating the degradation of mRNA [185,186]. The upregulation and expression of YTHDF2 have been observed in PH, and it has been found that inhibiting YTHDF2 can effectively hinder the proliferation of pulmonary arterial smooth muscle cells.

PASMC is generated by hypoxia. The study conducted by Huang et al. demonstrates that METTL14 exhibits direct recognition and binding affinity towards H3K36me3, which undergoes m6A alteration. Additionally, it has been observed that METTL14 is upregulated and expressed in pulmonary hypertension [187]. Inhibiting METTL14 has been found to effectively hinder the hypoxia-induced proliferation of PASMCs [188].

An increasing body of evidence suggests that lactate has a regulatory role in innate and adaptive immune cells, leading to notable alterations in gene expression through a distinct mechanism [89]. Lactate serves as a dynamic signal that governs the activity of immune cells and induces metabolic reprogramming to effectively alter their functionality. The modulation of immune responses and the significant biological significance of the immune system have been demonstrated by histone lactylation [189]. Lactate has the potential to significantly enhance the inflammatory response [59]. In the context of PH, it has been observed that an impaired immune system plays a crucial role in the process of pulmonary vascular remodeling. This impairment is characterized by an increased recruitment of inflammatory cells and the development of autoimmune dysfunction [190]. Lactylation modification plays a role in pulmonary hypertension by influencing the proliferation of pulmonary artery smooth muscle cells and vascular remodeling. The process operates by affecting mROS, gene expression, and inflammation, as shown in Figure 2E.

**Table 2:** Lactylation modification in cardiometabolic diseases.

Disease	Enzyme Regulation	Modification Sites	Cell Proliferation	Targets	References
Ischemic heart disease		H3K18la	BMDM	Lrg1, Vegf-a, IL-10	[142]
Heart failure	P300	Snail1	Cardiac endothelial cells	TGF-β	[146]
	p300/SIRT1	α-MHC K1897la	H9C2	Titin	[147]
Atherosclerosis		Mecp2k271la	ECs	Ereg	[152]
		H3K18la	BMDM	Arg1	[89]
		Sox10	VSMCs		[150]
	p300	H3Kla	VICs		[161]
Obesity, diabetes		FASN K673la	AML-12	FASN	[164]
		H2B(K6) la H4(K80) la	Hepatocyte	miR-155-5p	[166]
		H3K18la	ECs	FTO	[173]
		H3K18la	neutrophil	ROS	[174]
Hypertension		H3K18la H4K5la	PASMCs	Bmp5 Trpc5 Kit	[182]
		H3K18la	PASMCs	METTL3	[125,183]
		H3K18la	PASMCs	YTHDF2	[184]

This study summarizes lactylation modifications in ischemic heart disease, heart failure, atherosclerosis, obesity, diabetes, and pulmonary arterial hypertension, focusing on the modification sites, cell proliferation, and downstream targets.

## 6. Summary

The article provides an overview of lactate production and metabolism, the process of discovering and modifying lactylation, approaches to modifying specific sites, advancements in understanding non-histone lactylation, and explores the potential implications of lactylation in cardiometabolic disorders. Additionally, it discusses potential therapeutic strategies aimed at targeting lysine lactylation. Further investigation will be necessary in the foreseeable future to reveal the potential of lactoylation as a preventive and therapeutic target for various cardiometabolic diseases.

PTMs significantly facilitate the involvement of cells in a wide range of biological functions [191]. Several urgent challenges still need to be addressed in the research on lactylation modifications. First of all, post-translational modifications play a crucial role in epigenetic inheritance by affecting gene expression via the control of protein structure and function. Various post-translational modifications may have extensive crosstalk, and it is difficult to exclude other lysine acylations when studying Kla. Secondly, to investigate the correlations and interplay among various PTMs, numerous PTMs of target proteins have been detected using high-throughput mass spectrometry. However, there is a requirement to provide avenues for more comprehensive investigations in the field of proteomics. The metabolic regulatory mechanisms pertaining to lactate as the primary substrate for lactylation and its association with glycolysis have been partially established [89,106,192]. Next, the verification of non-enzymatic lactylation suggests that lactate is not the only substrate involved [90]. The metabolic pathway responsible for the conversion of lactate to lactyl coenzyme A, as well as the kinetics governing lactyl coenzyme A metabolism, remain unknown. Specifically, the focus lies on the identification of proteins involved in the transport of lactyl coenzyme A and the synthesis of lactyl coenzyme A. The investigation of the metabolic systems linked to lactyl coenzyme A has significant importance in comprehending the regulatory mechanisms preceding lactylation, as highlighted by Zhang et al. [89]. The comprehensive exploration of the downstream signaling pathways associated with lactylation has not been extensively studied, and there is ongoing debate on the epigenetic alterations related to lactylation. Finally, the processes of lactylation and acetylation have been investigated concurrently, employing a shared enzymatic “writer” and “eraser.” The enzymes responsible for lactylation have been identified by researchers, and these enzymes are also crucial for acetylation. No specific “writers” and “erasers” of lactylation changes have been identified. These “writers” and “erasers” are commonly observed in other processes and have been shown to have a similar function in lactylation modifications in later investigations. In particular, the “readers” of lactonization have not yet been studied, so there is still a huge research gap in the process of lactylation modification. Metabolic heart disease has become one of the leading global health killers due to high-sugar, high-fat diets, and environmental factors. Many researchers are diligently investigating the pathogenesis and treatment methods of metabolic heart disease. In 2023, Fan et al. first revealed the close relationship between lactic acid modification and heart failure after myocardial infarction, inspiring more researchers to delve into metabolic heart diseases. Currently, research in this area is not yet fully comprehensive, so researchers need to explore these directions further.

Autophagy is an evolutionarily conserved mechanism in which cytoplasmic elements are degraded in cells. It mediates organelle renewal and protein degradation and removes aged or damaged cytoplasmic components [193]. At the basic level, autophagy, as a major regulator of cardiac homeostasis and function, protects cardiomyocytes from stress injury, while excessive activation of autophagy aggravates myocardial injury and participates in the pathophysiological process of CMD [194]. Although the regulatory relationship between autophagy and lactylation modification has been demonstrated, the mechanism still needs to be further verified in metabolic heart disease [195,196]. Mitochondria are crucial for cellular metabolism, and their morphology and activity are cellular epigenetics. Mitophagy is an important part of mitochondrial quality control. Parkin-mediated mitophagy is a primary pathway for eliminating defective mitochondria in mammalian cells [197]. Deficiency of the Numb/Parkin pathway in prostate or lung adenocarcinoma leads to metabolic reprogramming that includes histone lactylation [198]. Mitochondria are extremely dynamic organelles that repeatedly fuse and fission to maintain homeostasis [199,200]. The lactylation of lysine 20 (Fis1 K20la) of the mitochondrial fission 1 protein (Fis1) was mediated by lactate. Fis1 K20la overexpression led to increased mitochondrial fission, which in turn caused ATP depletion, an excess of mitochondrial reactive oxygen species, and mitochondrial apoptosis. According to Susser et al., there is a direct correlation between mitochondrial length and macrophage phenotype, particularly when the macrophages move from a pro-inflammatory to a proresolving state [201]. In addition, Mao stated that intracellular hypoxia induces lactylation of mitochondrial proteins to inhibit OXPHOS [202]. A large amount of comprehensive research has been conducted on mitochondrial quality control, dynamics, and autophagy in cardiometabolic disorders. The mechanisms of lactylation and cardiometabolic disorders are also being gradually uncovered. Therefore, the regulatory processes of lactylation modification, mitochondrial function, and cardiovascular metabolic illnesses require a more detailed explanation.

## Figures and Tables

**Table 1:** Discovery of lactylation sites with the timeline.

Protein	Sites	Cell/Disease/Species	References
histone	6	*Botrytis cinerea*	[108]
histone	16	Brain/mouse	[106]
histone	16	*Trypanosoma brucei*	[107]
histone	14	Rice	[109]
histone	16	Maize	[110]
166	273	*Botryotinia fuckeliana*	[108]
257	387	*Trypanosoma brucei*	[107]
342	638	Rice grains	[109]
1014	2375	GC AGS cells/Human	[111]
523	983	*Toxoplasma gondii*	[112]
469	1458	*Frankliniella occidentalis*	[113]
955	1964	*Toxoplasma gondii*	[114]
420	636	*Phialophora verrucosa*	[115]
284	872	Tendon/Human	[116].
379	868	*Nannochloropsis oceanica*	[117]
139	215	Saccharum hybrid	[118]
960	2045	HCC/Human	[119]
751	3093	Microglia/Human	[120]
451	724	Lung/Human	[121]
469	1003	CIRI	[122]
	9275	HCC/Human	[123]
444	637	FHC SW480	[124]

Identification of lactylation sites together with the timeframe.

## Abbreviations

**Abbreviation**	**Full Name**
CVD	cardiovascular disease
CMD	cardiometabolic disorder
AS	atherosclerosis
PTM	post-translational modification
MVO2	myocardial oxygen consumption
PPCI	primary percutaneous coronary intervention
VSMCs	vascular smooth muscle cells
PH	pulmonary hypertension
ATP	adenosine triphosphate
NADH	nicotinamide adenine dinucleotide
LDH	lactate dehydrogenases
PDH	pyruvate dehydrogenase
MCTs	monocarboxylate transporters
SDLT	symptomatic lactate transport
HHF7	hyperinsulinemic hypoglycemia familial type 7
MCT1D	monocarboxylate transporter 1 deficiency
TME	tumor microenvironment
ETC	electron transport chain
H_2_O_2_	hydrogen peroxide
GPCRs	G protein-coupled receptors
PKA	protein kinase A
TAMs	tumor-associated macrophages
HPLC	high-performance liquid chromatography
MS/MS	tandem mass spectrometry
CBP	CREB-binding protein
HMGB1	high mobility group box-1
GC	gastric cancer
RCT	rotator cuff tendinopathy
HCC	hepatocellular cancer
CIRI	cerebral ischemia–reperfusion injury
METTL3	methyltransferase 3
ALDOA	fructose bisphosphate aldolase A
CENPs	centromere proteins
TCA	tricarboxylic acid
alpha-MHC	alpha-myosin heavy chain
CI/R	cerebral ischemia/reperfusion
Sox10	Sex-determining region Y (SRY)-related HMG-box gene 10
TNF-α	tumor necrosis factor-α
ECs	endothelial cells
MAPK	mitogen-activated protein kinase
AS	atherosclerosis
iNOS	nitric oxide synthase
Arg1	arginase-1
TLRs	Toll-like receptors
GSK3	glycogen synthase kinase 3
FOXO1	forkhead box protein O1
LPS	Lipopolysaccharide
lncRNA	long non-coding RNA
AGP	andrographolide
VICs	valve interstitial cells
LDL-C	low-density lipoprotein cholesterol
MPC	mitochondrial pyruvate carrier
iWAT	inguinal white adipose tissue
HTG	Huazhuo Tiaozhi Granule
DR	diabetic retinopathy
ROS	reactive oxygen species
mROS	mitochondrial reactive oxygen species
HIF-1α	hypoxia-inducible factor 1-alpha
PASMCs	pulmonary artery smooth muscle cells
